# Supplementary material for: The Conserved MAP Kinase MpkB Regulates Development and Sporulation without Affecting Aflatoxin Biosynthesis in Aspergillus flavus
Source: J Fungi (Basel). 2020 Nov 16;6(4):289. doi: 10.3390/jof6040289 (PMC7711526; doi:10.3390/jof6040289)
Supplement: Supplementary file 1 [file jof-06-00289-s001.zip › Afl_mpkB SuppTable1.pdf]

Supplemental Table 1. Primer sequences used in this study.

Primer	Sequence (5′ - 3′)	Purpose
AfupyrGF1	GGTGAAGAGCATTGTTTGAGGC	*A. fumigatus pyrG* marker
AfupyrGR1	AGTGCCTCCTCTCAGACAGAAT	〃
MpkBD5F	TGGCGGGTTACTTGGTGGTG	*mpkB* deletion cassette
MpkBD5R	*GGTGAAGAGCATTGTTTGAGGC*AAAAGTGGGGTGGGGCAGAA	〃
MpkBD3F	*AGTGCCTCCTCTCAGACAGAAT*GCTTGTCCATCGGGAAAGACTG	〃
MpkBD3R	ATCCTCGGCTCGGAGACTGG	〃
mpkB5NEST	GTTTTTGGGAGGGGAGACCC	〃
mpkB3NEST	GCTATGGTGCGGGTGTTGAA	〃
AflbrlAFRT	CACCGAGTCAATTGCGCCTC	*brlA* northern blot probe
AflbrlARRT	ACGGAATGGTGGGGACTGCT	*brlA* northern blot probe
AflmpkBFRT	GTTTGCGATTTTGGCTTGGC	*mpkB* northern blot probe
AflmpkBRRT	CGGGGAATAAGGGTTTTCCG	*mpkB* northern blot probe
AflnsdCFRT	GGCGAGCATGGGAAGTTACG	*nsdC* northern blot probe
AflnsdCRRT	TTCATCCATACGCTCGGGCT	*nsdC* northern blot probe
AflnsdDFRT	GGGCGAGTTTGGCATCAGTC	*nsdD* northern blot probe
AflnsdDRRT	TGTACCATAGCCGTTGGGGG	*nsdD* northern blot probe
AflsteAFRT	ATGATCGCAATGGAATGCCC	*steA* northern blot probe
AflsteARRT	GGTTCAGTGCCAAGCGGAAC	*steA* northern blot probe
AflveAFRT	CACCTTCGCATACAACGCCA	*veA* northern blot probe
AflveARRT	ACGGGCACACCGGTCAATAC	*veA* northern blot probe
AfupyrGPBF	CCTCCAAAGGATCGCTGGCT	*Afu_pyrG* southern blot probe
AfupyrGPBR	GCGTCCAATAGCCGATGCAG	*Afu_pyrG* southern blot probe
mpkBPBF	CAGCAACTTCCTCCCCAGGGA	*mpkB* southern blot probe
mpkBPBR	TCTGCCATCCACAAACCATCTG	*mpkB* southern blot probe
