# Supplementary figures and images for: The Conserved MAP Kinase MpkB Regulates Development and Sporulation without Affecting Aflatoxin Biosynthesis in Aspergillus flavus
Source: J Fungi (Basel). 2020 Nov 16;6(4):289. doi: 10.3390/jof6040289 (PMC7711526; doi:10.3390/jof6040289)

Supplemental Figure 1.

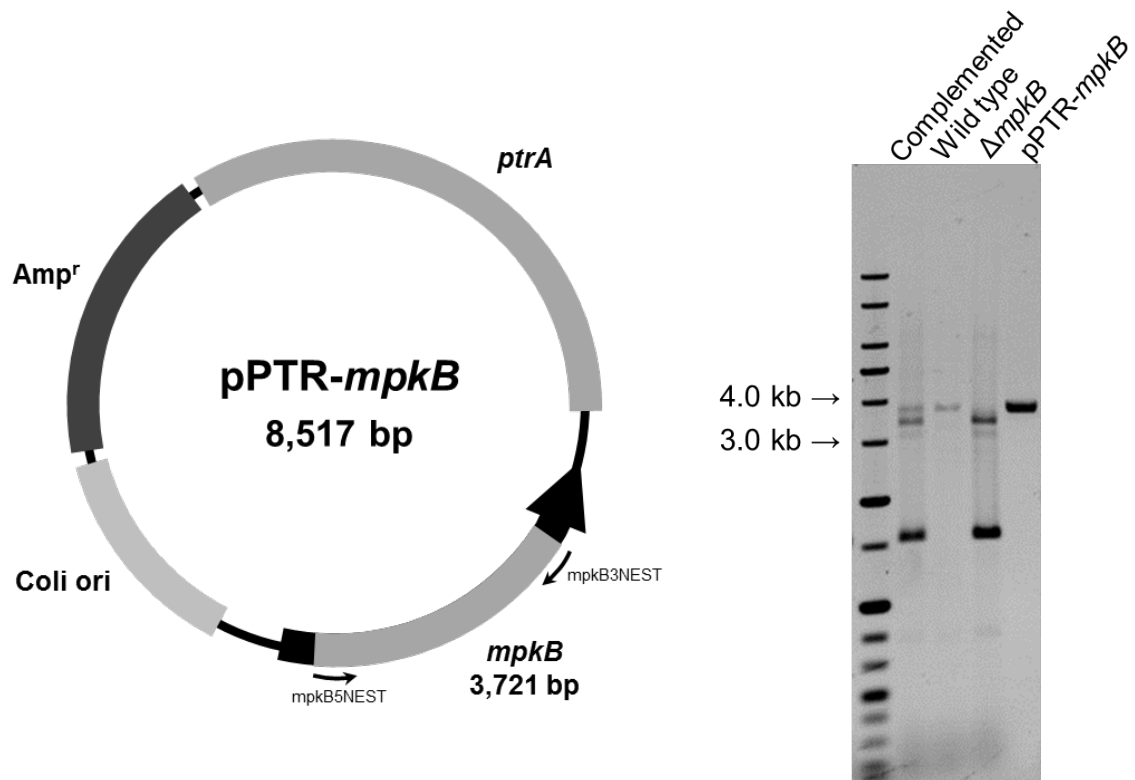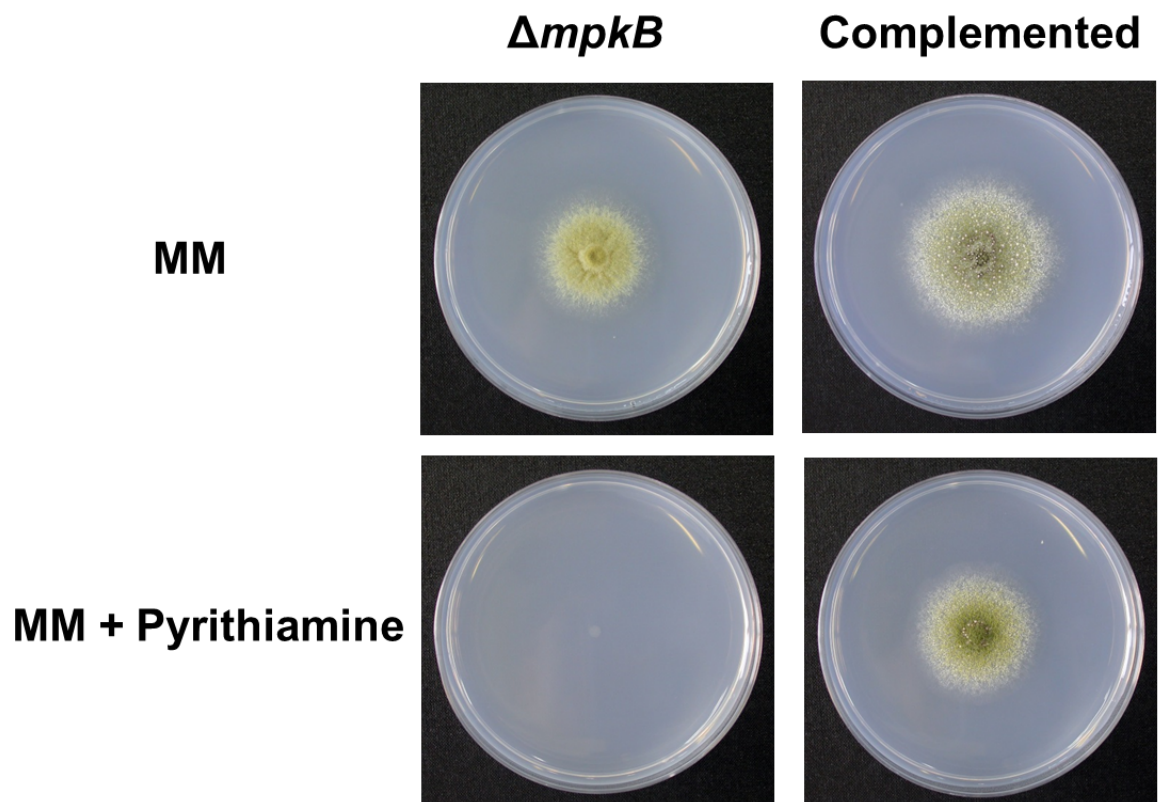

Supplement: Supplementary file 1 [file jof-06-00289-s001.zip › Afl_mpkB SuppFig1.pdf]

Supplemental Figure 2.

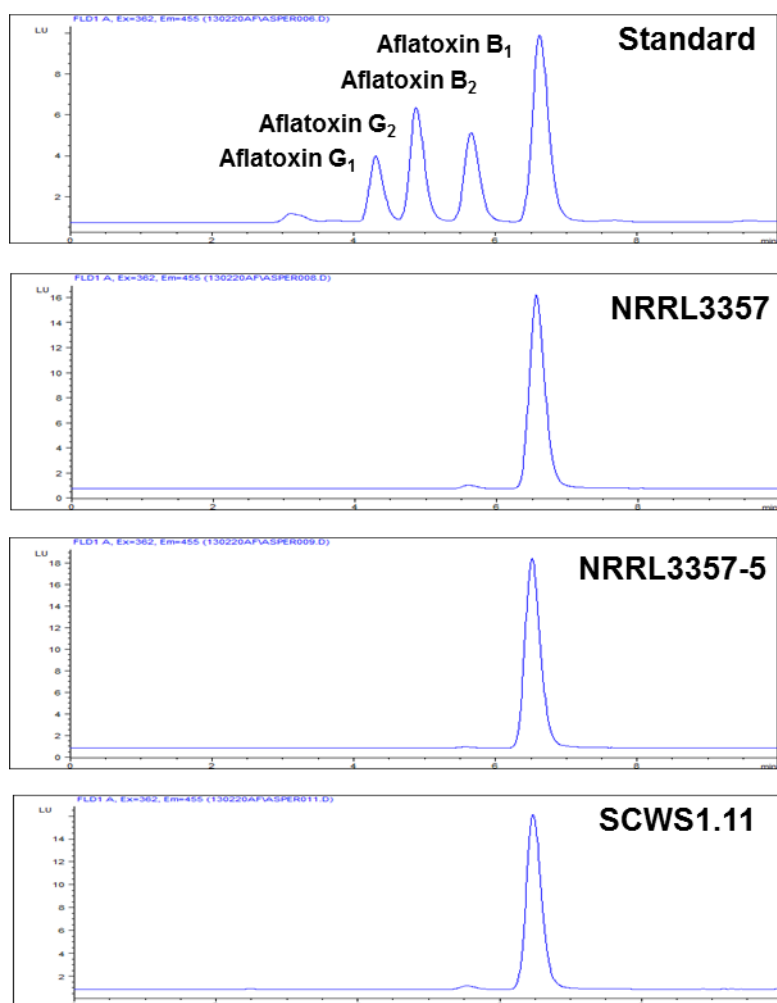

Supplement: Supplementary file 1 [file jof-06-00289-s001.zip › Afl_mpkB SuppFig2.pdf]
